# Pharmaceutical Composition of Valsartan: β-Cyclodextrin: Physico–Chemical and Characterization Anti-Hypertensive Evaluation

**DOI:** 10.3390/molecules15064067

**Published:** 2010-06-04

**Authors:** Carlos Eduardo de Matos Jensen, Robson Augusto Souza dos Santos, Angelo Márcio Leite Denadai, Cynthia Fernandes Ferreira Santos, Aline Nardoni Gonçalves Braga, Rubén Dario Sinisterra

**Affiliations:** 1 Departamento de Química, ICEx, Universidade Federal de Minas Gerais, Avenida Pres. Antônio Carlos 6627, 31270-901, Belo Horizonte, Brazil; E-Mail: cej.jensen@gmail.com (C.E.M.J.); 2 Departamento de Fisiologia e Biofísica, ICB, Universidade Federal de Minas Gerais, 31270-901 Belo Horizonte, Brazil; E-Mails: robsonsant@gmail.com (R.A.S.); cynthiaff.santos@gmail.com.br (C.F.F.S.); losartan04@yahoo.com.br (A.N.G.B); 3 Campus Centro Oeste Dona Lindu, Universidade Federal de São João del Rei, R. Sebastião Gonçalves Coelho, 400, 35501-296, Divinópolis, Brazil

**Keywords:** valsartan, β-cyclodextrin, intrinsic dissolution, ROESY, anti-hypertensive evaluation

## Abstract

Valsartan, a water-insoluble drug, is mainly used in the treatment of hypertension albeit with reduced oral bioavailability. The aim of work was to develop a valsartan:β-cyclodextrin (VAL:β-CD) pharmaceutical composition in order to improve its water solubility and bioavailability. The VAL:β-CD complexes were prepared by the kneading, solid dispersion and freeze-drying methods, of which the freeze-drying method (FDY) was found to be the best to prepare an inclusion complex. A physical mixtyure PM was also prepared. Complexes were characterized by thermal analysis, Fourier transformed- infrared (FTIR) spectroscopy, Powder X-ray diffractometry, intrinsic dissolution and NMR (2D-ROESY). Phase-solubility analysis showed A_L_-type diagrams with β-cyclodextrin (β-CD). Microcalorimetric titrations suggested the formation of 1:1 inclusion complex between VAL and β-CD. The apparent stability constants *K*_1:1_ calculated from phase-solubility plots were 165.4 M^-1^ (298 K), 145.0 M^-1^ (303 K) and 111.3 M^-1^ (310 K). *In vivo* experiments in rats showed that reduction in arterial pressure for the FDY complex is better than with valsartan used alone. The better activity of FDY can be attributed to the higher solubility of valsartan after inclusion in the cyclodextrin cavity, as suggest by the intrinsic dissolution studies.

## 1. Introduction

**Figure 1:**
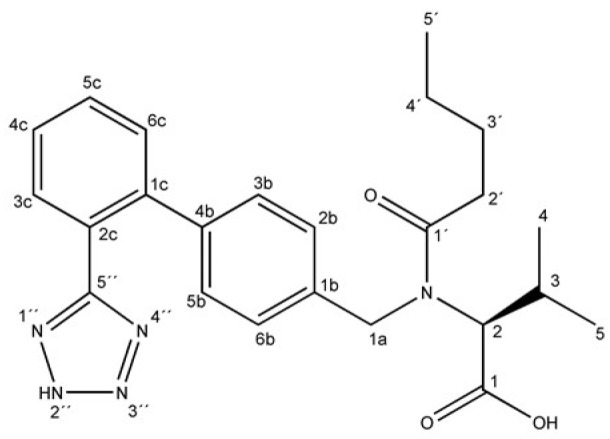
VAL or 3-methyl-2-[pentanoyl-[[4-[2-(2H-tetrazol-5-yl)phenyl]phenyl]methyl]-amino] -butanoic acid [CAS number: 137862-53-4].

Hypertension is one of the most prevalent chronic adult illnesses today and cannot be cured, but it can be controlled. The pharmacological treatment for control of hypertension utilizes various drug therapies, single doses or associations of diuretics, beta-blockers, calcium channel blockers, angiotensin converting enzyme (ACE) inhibitors and angiotensin II receptor (AT1) antagonist (ARA) [1,2]. Valsartan (VAL, Figure 1) is one of the angiotensin II receptor (AT1) antagonists recommended for treatment of hypertension, post-myocardial infarction or congestive heart failure. VAL is administered at a dose of 80 mg or 160 mg per day. Other drugs in the same group include losartan, irbesartan, olmesartan and candesartan [3,4].

ARAs have different chemical structures derived from imidazole-5-acetic acid with a common tetrazole-biphenyl structure. The differences between ARAs are related to structural features that lead to changes of their acid dissociation constants (pKa), partition coefficients (LogP), as well as absorption and bioavailability. After oral administration, only 25% of VAL (Figure 1) is absorbed, food intake being one of the possible causes for the low bioavailability [5]. Other drugs from the ARA group have bioavailabilities ranging between 33% (losartan) and 60–80% (irbesartan) [6,7]. The low bioavailability of VAL is associated with its poor water solubility. Despite the reduced water solubility VAL is freely soluble in alkaline solution as the corresponding salt.

According to the Biopharmaceutics Classification System (BCS) aqueous solubility and permeability are the most important variables affecting drug bioavailability. VAL, along with telmisartan, irbesartan and candesartan, is classified as Class II, that is drugs that have low solubility and high permeability characteristics after oral administration. Losartan is included in Class III, where the drugs have high solubility and low permeability [8]. The use of cyclodextrins is one of the pharmaceutical strategies available to circumvent these drawbacks, as they can be used as complexing agents to increase the aqueous solubility of hydrophobic drugs and to increase their bioavailability and stability [9,10,11,12].

Cyclodextrins (CDs) are cyclic oligosaccharides consisting of (α-1,4)-linked α-D-glucopyranose units that contain a lipophilic internal cavity and a hydrophilic outer surface resembling the shape of a truncated cone. The hydroxyl functions of the glucose are orientated to the exterior of the cone, where primary hydroxyl groups are in the narrow edge of the cone and the secondary hydroxyl groups at the wider edge. The inner cavity is lined by skeletal carbons and ethereal oxygens of the sugar residues, which gives it a lipophilic character. These characteristics allow CDs to form inclusion complexes with a variety of host-guest molecules of suitable polarity and size. Their use has been extensively exploited to improve the pharmaceutical properties of numerous drugs such as water solubility, stability, physicochemical incompatibilities, oral absorption and controlled release in order to modulate biological activity [9,11,13,14,15].

The main objective of the present work was to use the host:guest strategy to obtain a more efficient VAL pharmaceutical employing cyclodextrins. The interaction between the VAL:β-CD binary systems was studied in solution using the phase solubility method. VAL:β-CD solid systems in equimolar ratio were prepared through physical mixing, and the solid dispersion, kneading and freeze-drying methods. All the adducts were characterized by Thermal Analysis (TG/DTG and DSC), Fourier Transform Infrared Analysis (FTIR), ROESY-NMR spectroscopy and intrinsic dissolution experiments. *In vivo* experiments in rats were performed to evaluate the anti-hypertensive properties of the resulting VAL:β-CD complexes.

## 2. Results and Discussion

**Figure 2:**
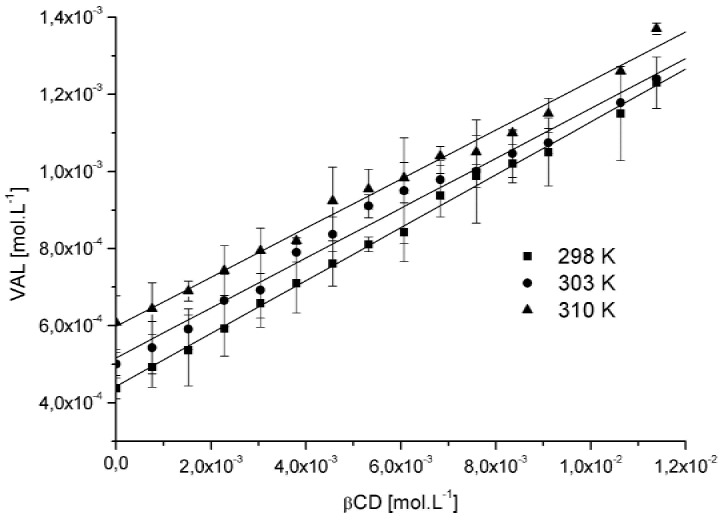
Phase solubility diagram of VAL:β-CD inclusion complex at 298, 303 and 310 K.

The phase solubility diagrams obtained in the study with β-CD and VAL are depicted in Figure 2. Analyzing the phase solubility profiles it is possible to observe the increase of VAL solubility in the system due to molecular interaction with β-CD. VAL formed soluble complexes with β-CD in water showing a typical A_L_-type solubility diagrams [16] where the inclusion stability constant (K_c_), calculated according to Equation (1) and summarized in Table 1, indicate a decrease of K_c_ when the temperature increase, as expected for an exothermic process.

Similar temperature effects on the stability constants were found by Brunella, *et al.* and Karathanos *et al*. [9,12,17,18]. The K_c_ values indicate a moderate host:guest affinity by VAL due to the hight molecular weight and lower water solubility, which hindered the insertion of molecule inside the β-CD cavity [9,19]. The integrated form of the van’t Hoff equation (1) permits calculation of entropy and enthalpy changes, from variations of the K_c_ in different temperatures:

(1) $$\ln K_{c}=-\frac{\Delta H^{0}}{RT}+\frac{\Delta S^{0}}{R}$$

**Figure 3:**
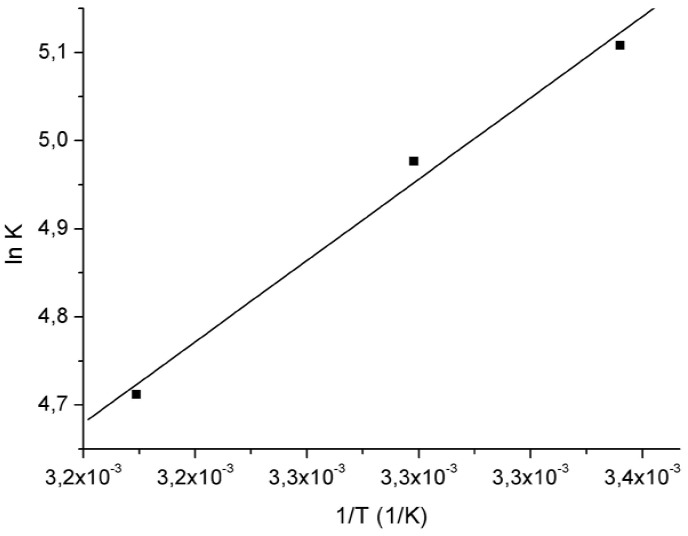
van’t Hoff plots (ln K *versus* 1/T) for VAL:β-CD complexation, determined by solubility diagram experiments.

The van’t Hoff plot for the VAL:β-CD complex is a linear function between K_c_ and the inverse of absolute temperature (1/T), as shown in Figure 3. The thermodynamic parameters Δ*H*, Δ*S* and Δ*G* for the host:guest compound were calculated and are shown in Table 2. The enthalpy change (∆*H*) is -24.90 kJ·mol^-1^, which is relatively small, indicating that the VAL:β-CD interaction is an exothermic process and typical of low energy interactions, such as hydrophobic van der Waals interaction due to the displacement of water molecules from the cavity of the βCD. The change of entropy (Δ*S*) is -0.041 J·mol^-1^·K^-1^ and can be explained considering that complexation process causes a decrease in translational and rotational degrees of freedom of the VAL included as compared with the free ones. The standard Gibbs energy change (∆*G*) is -12.45 kJ·mol^-1^, calculated through enthalpy and entropy changes, shows the spontaneous formation of VAL:β-CD inclusion complex in aqueous solution [9,12,15,18,20].

Evidence of interactions between VAL and β-CD in the solid state can be obtained using thermal analysis. When guest molecules are included in CD cavities, their melting, boiling, glass transition and sublimation points shift to different temperatures or disappear [21].

In order to confirm the average stoichiometry of complex in solution, isothermal calorimetric titrations – ITC – were performed. Microcalorimetric titration allows simultaneous determination of the enthalpy, equilibrium constant and stoichiometry from a single titration curve, through a least square non-linear adjusted line derived from the Wiseman isotherm [22], according to the equation (2):

(2) $${\left(\frac{dQ}{d{\left[Val\right]}_{tot}}\right)}_{P}=\Delta H^{o}Vo\left[\frac{1}{2}+\frac{1-X_{R}-r}{2\sqrt{{\left(1+X_{R}-r\right)}^{2}-4X_{R}}}\right]$$

This above equation relates to the stepwise change in heat of the system normalized with respect to moles of valsartan added per injection (dQ/d[Val]_t_)P at constant pressure, to the absolute ratio of ligand to receptor concentration (XR = [Val]_t_/[β-CD]_t_) at any point during the course of the titration. The parameter ΔH is the molar enthalpy of binding, V_o_ is the initial volume of the β-CD solution (1.5 mL) in the titration cell and r is composition variable 1/[ β-CD]_t_.K_c_.

**Figure 4:**
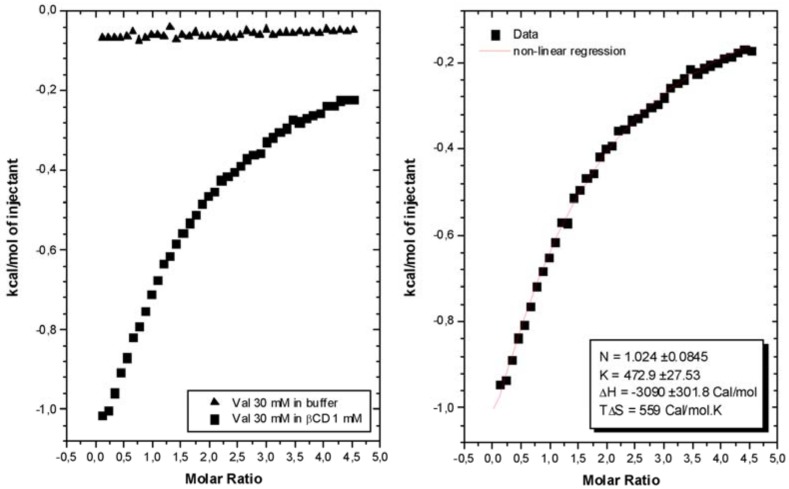
Isothermal titration microcalorimetry of VAL - βCD interactions.

The experimental data for the titration of valsartan 30 mM in buffer (blank experiment) and valsartan 30 mM in βCD 1 mM solution, at temperature of 298 K, is shown in Figure 4. The thermodynamic properties were calculated after subtraction of the blank curve as shown in the figure, using the “one binding site” model. The non-linear regression showed an equivalence point at a molar ratio of approximately 1.0. These data and the relative size of the molecules suggest strongly a 1:1 stoichiometry. It was observed that interaction between valsartan and β-CD is principally exothermic. The enthalpy changes could be attributed to binding of enthalpy-rich water molecules released from the cyclodextrin cavity with bulk water molecules, in agreement with the literature [23], and/or the formation of cooperative van der Waals interactions between guest and the cyclodextrin cavity through the hydrophobic valsartan moiety.

**Figure 5:**
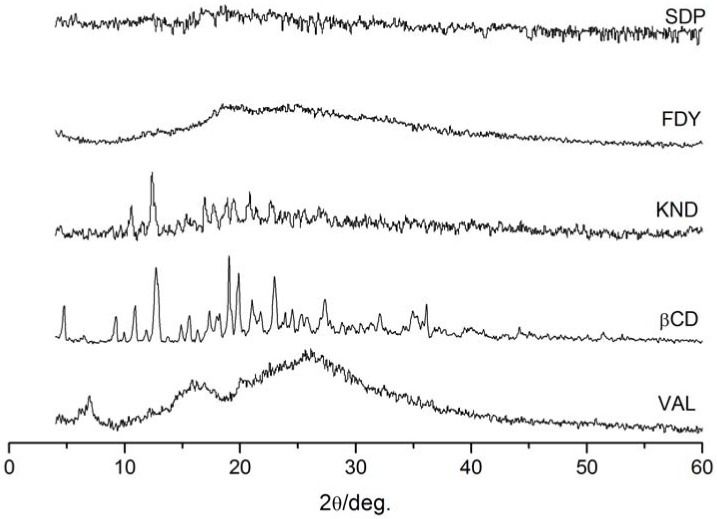
XRD diffractogram of VAL, β-CD and the complexes prepared by the solid dispersion (SDP), freeze-drying (FDY) and kneading (KND) methods.

X-ray powder diffraction patterns of VAL, β-CD and the corresponding complexes of VAL with β-CD are shown in Figure 5. In the X-ray diffractogram of VAL its possible to characterize the powder as a semi-crystalline product. No diffraction peaks relevant to VAL were detectable in all the systems with β-CD. Complete drug amorphization was instead observed in SDP and FDY. For KND the presence of free crystalline β-CD was revealed by peaks at 12.4° and 19.0° 2θ. The diffractogram of the KND differs much from that of SDP and FDY as well as VAL and β-CD, indicating no complex formation. In the literature there is described a complex formed by VAL and β-CD as a crystalline material, with peaks in 12.7, 10.8 and 19.8 2θ [24].

The TG/DTG curve results for VAL and β-CD are not shown. Analyzing the TG curve for β-CD, we can observe a weight loss in the range of 30 °C to 95 °C, which corresponds to the loss of eight water molecules present in the β-CD internal cavity. Next, there is a thermal stability period up to approximately 350 °C, followed by total mass loss at 700 °C, indicating complete decomposition of the β-CD in accordance with the literature [21,24,25]. In regards to the studies of complexes between β-CD and VAL prepared using the kneading method, solid dispersion, as well as physical mixture, there was no solid residue generation at the end of analyses. This indicates complete decomposition of the substances. During the freeze-drying method, there was a solid residue generation corresponding to 7.84% (± 0.093) of the whole sample. This residue is possibly related to potassium oxide as result from potassium hydroxide use for pH adjustment of the freeze-drying solution.

**Figure 6:**
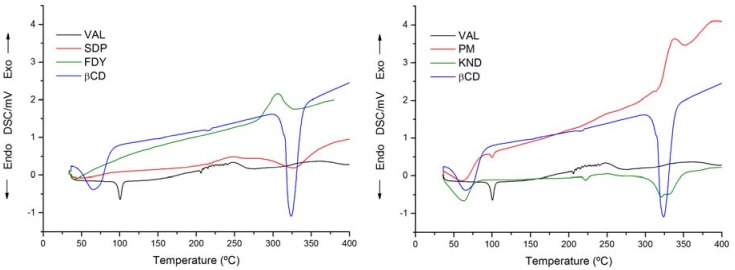
DSC curves for β-CD, VAL, the complexes prepared by the solid dispersion (SDP), freeze-drying (FDY), kneading (KND) methods and the physical mixture (PM).

DSC was another technique employed for the characterization of the complexes. The DSC thermal profiles for the samples are represented in Figure 6. DSC curve for VAL shows an endothermic peak at 100.5 °C related to drug melting point. A weak association is show only for physical mixture (PM), where the melting peak is observed at 100.5 °C in contrast with the observations for the SDP, FDY and KND compounds [26]. The absence of a melting peak of VAL in the solid was taken as an indication that the drug is included in β-CD cavity, leading to a reduction in the overall crystallinity of the system.

**Figure 7:**
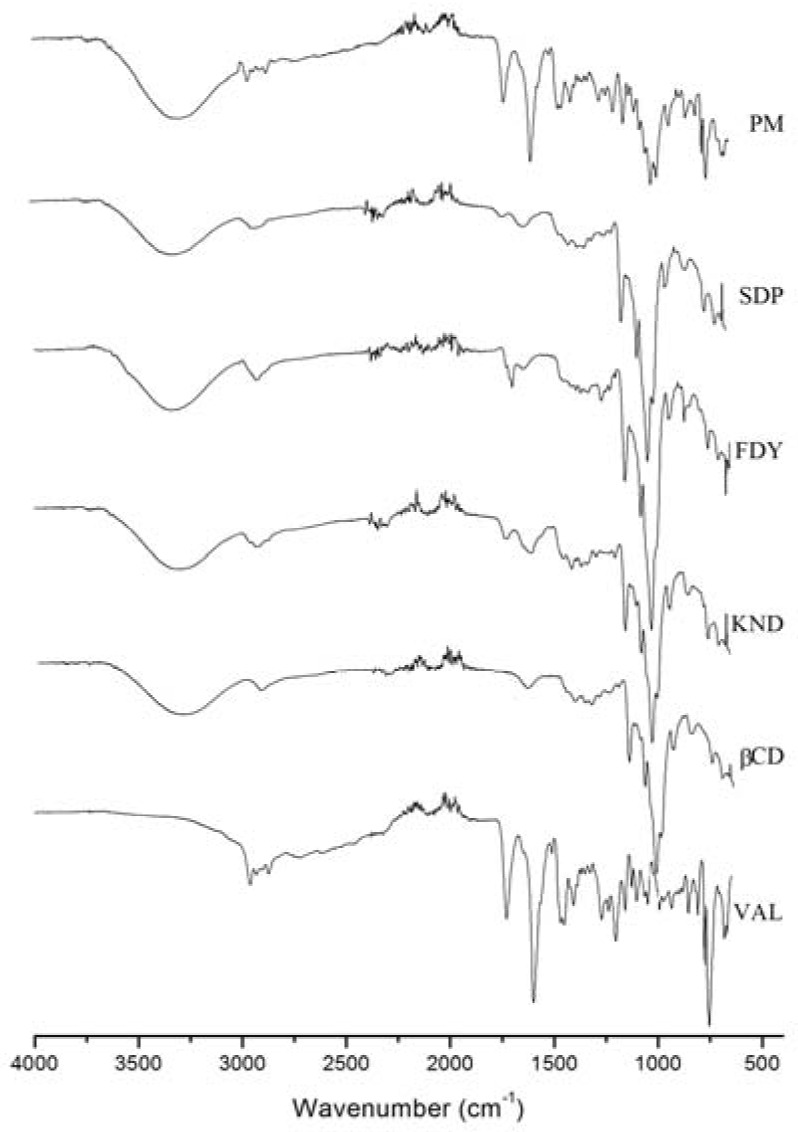
FTIR spectra for β-CD, VAL, the VAL and β-CD complexes prepared by solid dispersion (SDP), freeze-drying (FDY), kneading (KND) methods and the physical mixture (PM).

The IR spectra, shown in Figure 7, could reveal important changes in some vibrational modes as a consequence of the host:guest interactions. The VAL proportion was the same in all complexes – 24.8% w/w (± 2.57). The spectra of the inclusion complex FDY, KND, SDP and the PM were dominated by the vibrational bands of the cyclodextrin molecule since there are seven repeating glucose units in the β-CD molecule. In the inclusion compounds, when the molecule is included into the hydrophobic β-CD cavity no significant displacement of signals in the FTIR spectra is Expected. The host:guest interaction is mediated by weak forces between molecules, such as hydrogen bonding, hydrophobic interactions as van der Waals forces, *etc*.

VAL has shown the presence of two strong stretching absorption bands around 1,731 cm^-1^ and 1,600 cm^-1^, corresponding to the carbonyl group (amide and carboxylic acid) contributions. Analyzing the SDP, FDY and KND FTIR spectra we can observe low intensity of these stretching ν_(C=O)_ upon host:guest interaction in contrast with the PM FTIR spectra where no perceptible changes were noted. Moreover, the ν_(C=C)_ aromatic ring deformation at 1,455 cm^-1^ of VAL also diminished in intensity after the association with β-CD but was explicit in the PM. Based on the above results it is reasonable to suggest that there is a formation of new supramolecular compound after association of VAL with β-CD [17,27].

The inclusion complex of VAL with β-CD has been described in the literature [28]. The IR spectra of this complex, prepared in the 1:1 molar ratio, showed a band shift for the carboxyl carbonyl group of valsartan, which in the physical mixture appears at 1,732 cm^-1^ and in the complex at 1,734 cm^-1^. Moreover, the authors explain that the band shift characteristic for the amide carbonyl group in the physical mixture appears at 1,646 cm^-1^, while in the complex it appears at 1,642 cm^-1^. Others signals also change and the authors concluded that these alterations confirm that an inclusion complex was formed. Our work is in accordance with these results. 

**Figure 8:**
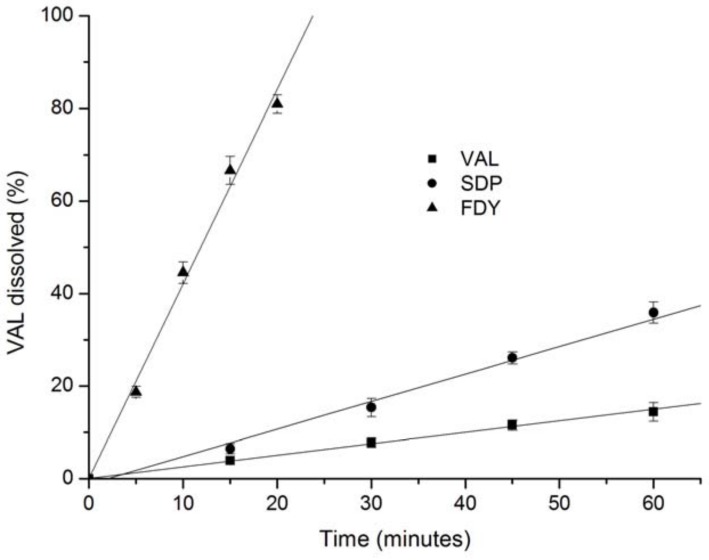
Intrinsic dissolution profiles for VAL and complexes prepared by the solid dispersion (SDP) and freeze-drying (FDY) methods.

Intrinsic dissolution is a pharmaceutical method which utilizes a compressed disc of known area (with a constant surface) eliminating surface area and surface electrical charges as dissolution variables. The dissolution rate observed is specific for each solid product in a given solvent under the determined experimental variables. The result is generally expressed as mg dissolved per minute per centimeter squared [29]. Based on these results, it is possible to predict problems due to dissolution rate of the drug [30]. Intrinsic dissolution studies were performed in order to study the effect of inclusion process on the dissolution of VAL. Figure 8 show the plot of VAL and the SDP and FDY VAL:β-CD complexes dissolved *versus* time at pH 6.8. The data were normalized to percentage of VAL released *versus* time. The linearity was higher than 0.99 (Table 3) and the calculated intrinsic dissolution shows a small Relative Standard Deviation (RSD), indicating good reproducibility. It is interesting to note a higher dissolution rate of at least 16 times and 2.4 times for the FDY and SDP complexes, respectively, when compared to the free VAL dissolution rate. These results are very relevant as the increased VAL dissolution rate could increase its bioavailability [8].

^1^H-NMR spectroscopy in solution is on the most effective methods for studying cyclodextrin inclusion complexes [31,32,33,34,35,36,37]. Two dimensional (2D) NMR is known to allow the observation of inter- and intra-molecular interactions [38]. The presence of NOE cross-peaks between protons from the host:guest species indicates spatial contacts of less than 5 Å. In order to get more information, we used 2D ROESY to study the host guest compounds, analyzing cross-peaks involving the protons (H3 and H5) inside the β-CD cavity. 

**Figure 9:**
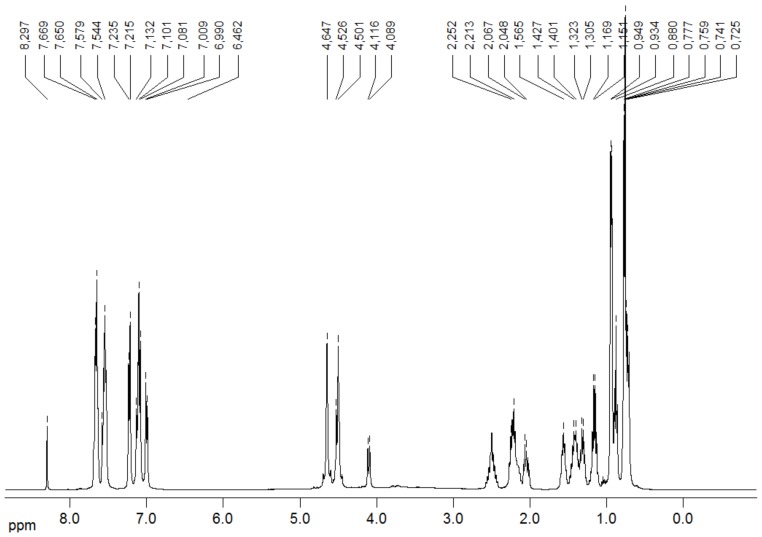
^1^H-NMR spectrum of valsartan in DMSO-d_6_ at 400 MHz at 27 °C.

**Figure 10:**
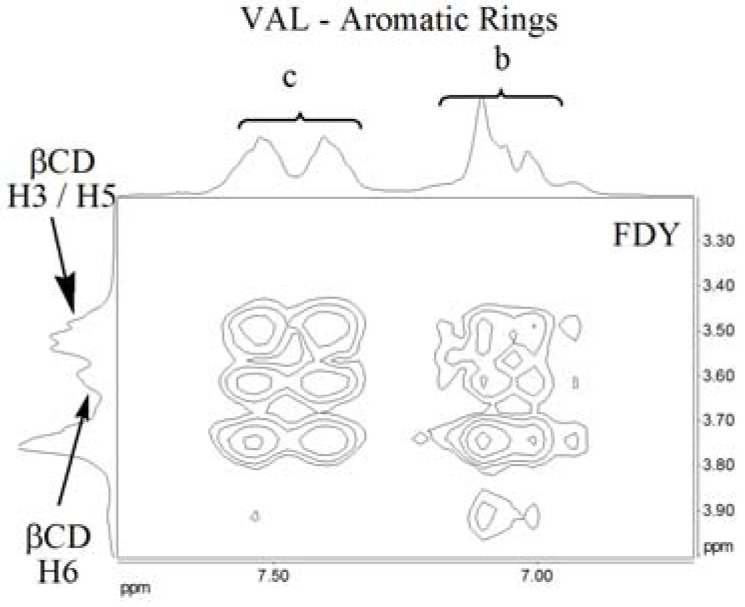
Partial contour map of the 2D-ROESY in DMSO-d_6_ at 400 MHz for the VAL:βCD complex prepared by freeze-drying (FDY) method.

Figure 9 shows the ^1^H-NMR spectrum of valsartan in DMSO-d_6_ and the Figure 10 shows the partial contour map of 2D-ROESY experiments in DMSO-d_6_ for inclusion complexes prepared by the FDY method. The results (data not shown) demonstrate that there are no cross-peaks between VAL and β-CD in the complex resulting from KND and SDP. Such an interaction can only be observed in the complex prepared by the FDY method (Figure 10). The correlation occurs between the hydrogens (H3 and H5) located inside the β-CD cavity with the aromatic hydrogens of the “b” (δ 7,544–7,669 ppm) and “c” VAL rings (δ 6,462–7,235 ppm). Another correlation can be observed between the aromatic “b” and “c” rings of VAL and H6 of β-CD, confirming the formation of the inclusion complex. These results could suggest the presence of two valsartan conformers at room temperature.

**Figure 11:**
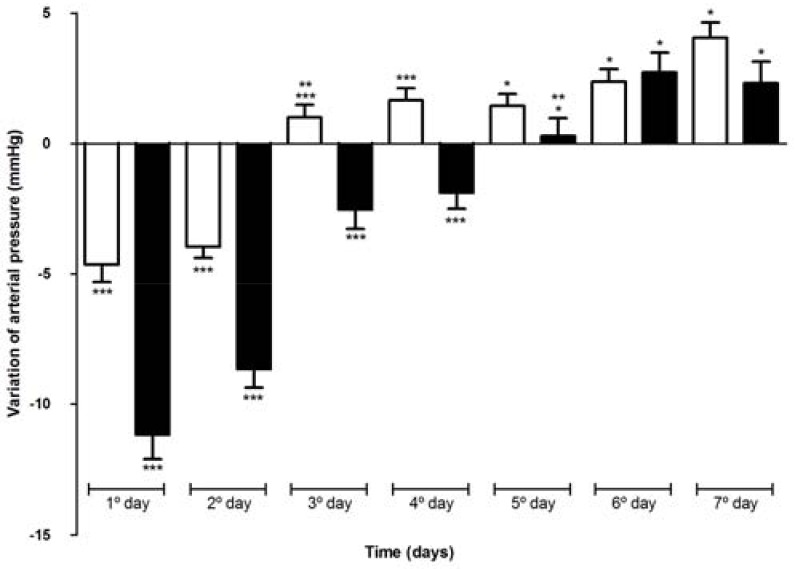
Arterial pressure variation of Wistar rats after one oral dose of VAL (white bars) or FDY (black bars). * no significant difference between VAL and FDY, ** p > 0.05 in comparison to control period, *** p < 0.0001 comparing VAL and FDY in the same day.

Figure 11 shows the variation of arterial pressure in Wistar rats after oral administration of VAL or FDY complex. As shown, the single oral administration of 1.14 mg.kg^-1^ of VAL in hypertensive rats produced a hypotensive effect. VAL alone significantly reduced blood pressure for about two days. The most significant reduction in arterial pressure occurred in the first day and was -4.65 ± 0.67 mmHg compared with the control period (p < 0.05) and the mean reduction in arterial pressure in these two days was -4.00 ± 0.36 mmHg. On the third day the values of reduction in arterial pressure were not significantly different from those observed in the control period (p > 0.05). On the other hand, the FDY produced a significant reduction in arterial pressure after forty minutes and in the first day this reduction was -11.16 ± 0.92 mmHg compared with control period (p < 0.05). The hypotensive effect of FDY was significant for four days compared with control period (p > 0.05) and the mean reduction in arterial pressure in these four days was -5.17 mmHg ± 0.38 mmHg. The heart frequency was measured (data not shown) and did not change significantly during the experiment. These results could be due to the higher solubility of the FDY complex that causes a faster and prolonged action of VAL.

## 3. Experimental Section 

### 3.1. General

VAL (M_w_ = 435.53) was purchased from Hetero Labs Limited (Hyderabad, India) and β-CD (Mw = ~1135.01) was purchased from Sigma-Aldrich (Milwaukee, WI, USA) and used as received. All other chemicals and solvents were of pharmaceutical or analytical reagent grade. The water used in the experiments was ultrafiltered with Milli Q plus equipment from Millipore® (Billerica, MA, USA).

### 3.2. Phase solubility studies

The VAL phase solubility studies were carried out according to the method previously reported by Higuchi and Connors [16]. Briefly, excess amounts of VAL (15 mg) were added to water (2 mL) containing various concentrations of β-CD (0 to 0.01215 mol·L^-1^). The suspensions were shaken at 298, 303 and 310 K for three days. After reaching equilibrium, the samples were filtered through a 0.45 μm membrane filter (Millipore^®^) and suitably diluted in 0.01 mol·L^-1^ NaOH. VAL concentration was determined spectrophotometrically (HP UV/Vis spectrophotometer, model 8453, Hewlett Packard, Palo Alto, CA, USA) at 225 nm. The stability constants, K_c_, were calculated from the phase solubility diagrams assuming the 1:1 stoichiometry, according to the equation:

(3) $$K_{1:1}=\frac{slope}{S_{o}(1-slope)}$$

where S_0_ is VAL solubility in the absence of β-CD.

### 3.3. Microcalorimetric measurements

Calorimetric titrations were carried out in duplicate with a VP-ITC Microcalorimeter from Microcal at 298.15 K. The ITC instrument was previously calibrated electrically and chemically. Each titration experiment consisted of 41 successive injections of valsartan (30 mM) dissolved in phosphate buffer solution into the reaction cell charged with of β-CD (1.5 mL, 1 mM) in phosphate buffer solution, with time intervals of 540 s. The first injection of 1 μL was discarded to eliminate diffusion effects of material from syringe to cell calorimetric. The subsequent were injected at constant volume of 5 μL of valsartan. The time of injection was 2 s. The β-CD concentration in the calorimeter cell varied from 1 to 0.86 mM and the concentration of the valsartan from 0.0 to 4.3 mM. The raw data were analyzed by the software supplied with the calorimeter (Microcal Origin 5.0 for ITC), next the subtraction of blank experiment (dilution of valsartan in phosphate buffer solution).

### 3.4. Preparation of inclusion complexes

The VAL:β-CD inclusion complex was prepared using three different methodologies, namely kneading (KND), solid dispersion (SDP), freeze-drying (FDY), which are described below. The 1:1 molar ratio was considered based on previous solubility studies. A physical mixture (PM) was also used. The powder obtained was stored at 4°C until physicochemical characterization.

#### 3.4.1. Kneading method

In order to prepare the complex using the kneading method, the β-CD and VAL were transferred to a glass mortar, mixed and homogenized with water in the proportion of 1:4 (solid-water) to produce the slurry, which was kneaded for 45 min. Following this period, the material was dried at 40 °C for 24 h.

#### 3.4.2. Solid dispersion

Complexes were also prepared by the solid dispersion method. Initially β-CD (200 mg) was dissolved in water at concentration of 1.67 w/v%. VAL (77 mg) was dissolved in ethyl alcohol (1 mL) and added to the β-CD solution in order to allow complexation. This suspension obtained was kept in an ultrasonic bath for 10 min, frozen using liquid nitrogen and freeze-dried for 24 h.

#### 3.4.3. Freeze-drying 

VAL (77 mg) was dispersed in water and the pH was adjusted to 6.0 with 0.1 mol·L^-1^potassium hydroxide. The drug was soluble under these conditions because it is a weak acid, its pKa being 4.73. The solution containing VAL was mixed with a solution containing β-CD (200 mg) and stirred at room temperature for 24 h. Then, the solution was freeze-dried and the powder obtained was stored at 4 °C. The freeze-dryer used was a Thermo Savant Modulyo D Freeze Dryer-115 instrument (Ramsey, MN, USA).

#### 3.4.4. Physical mixture

For comparison reasons, 1:1 physical mixtures were prepared Briefly, β-CD (200 mg) was transferred to a glass mortar together with VAL (77 mg) and mixed for 5 min.

### 3.5. Powder X-ray diffractometry

X-Ray powder diffraction patterns were recorded at room temperature using a Rigaku Geigerflex 2037 from Rigaku Corp. (Tokyo, Japan). The measurement conditions were as follows: Co-filtered, Cu Kα radiation, scanning speed of 4θ per min over a 2θ range of 4° to 60°.

### 3.6. Thermal analyses

Differential Scanning Calorimetry (DSC) curves were produced in triplicate in a DSC-50 instrument (Shimadzu Co., Kyoto, Japan) using the following conditions: dynamic nitrogen atmosphere (50 mL·min^-1^), heating rate of 10 °C·min^-1^. Samples were weighted out accurately and submitted to further heat scanning from 25 °C to 500 °C in a sealed aluminum pan with a capacity of 40 μL. An empty sealed aluminum pan was used as reference. The equipment was periodically calibrated with indium (99.98%, m.p. 156.65 °C, Sigma-Aldrich, Milwaukee, WI, USA). Data analysis was carried out using TA Analysis Software.

### 3.7. Fourier transformed-infrared (FTIR) spectroscopy

Infrared spectra covering the range of 4,000–400 cm^-1^ were obtained with a FTIR spectrometer (Spectrum one, Perkin Elmer, Waltham, MA, USA). The spectra were an average of 32 scans at a resolution of 4 cm^-1^.

### 3.8. Nuclear Magnetic Resonance (NMR) experiments

NMR spectra were recorded on a DRX-400 Avance 400 MHz spectrometer (Bruker-Biospin, Rheinstetten, Germany) at 300 K. DMSO-d_6_ (isotopic purity at least 99.5%) was purchased from Aldrich and used as solvent, and tetramethylsilane (TMS) as internal standard (δ 0.0). The solutions were transferred to NMR tubes with 8 inches in length and 5 mm in external diameter. One-dimensional NMR experiments (^1^H and ^13^C) were performed with a 5 mm dual probe (^1^H/^13^C) using inverse detection with z-gradient coil. The intermolecular interaction between β-CD and VAL was monitored by ^1^H-NMR and ^1^H-^1^H rotating-frame nuclear overhauser spectroscopy, 2D-ROESY (500 ms spin lock). The water suppression was performed using the WATERGATE technique [38,39,40]. Data were processed using the software XWIN NMR, 3.1 (Bruker-Biospin, Rheinstetten, Germany) and its edited with Mestre C®, version 4.9.9.6.

### 3.9. Intrinsic dissolution

The dissolution test was conducted under sink conditions in phosphate buffer (900 mL, 0.05 mol·L^-1^, pH 6.8) at 37 ± 0.5 °C and rotational speeds of 100 rpm. Each dissolution test was performed at least in triplicate [29]. The dissolution system was fitted with ERWEKA DT800 (Distek Inc., NJ, USA) and a HP 89092A 7-channel peristaltic pump (Agilent Technologies Italia Spa., Roma, Italy). The aliquots collected containing VAL were filtered using a 0.45 μm filter (Millipore^®^), mixed with 0.01 mol·L^-1^ NaOH and analyzed in a spectrophotometer at 225 nm. FDY, SDP complexes and VAL discs were prepared compressing powder (200.0 mg) in a Perkin Elmer hydraulic press (Waltham, Massachusetts, USA) for 1 min under 3 t compression force, using a 8 mm punch. The surface area exposed was 0.5 cm^2^ and the disk distance from vessel bottom was 2.54 cm. Aliquots of VAL from the mixture prepared by the freeze-drying (FDY) method were automatically collected every 5 min until 20 min. For SDP and VAL the aliquots were collected every 15 min until 60 min. The results were normalized to the percentage of VAL released, a linear regression of VAL released *versus* time was plotted and the intrinsic dissolution rate of the drug was determined in mg per minute per cm^2^ from the slope of the regression line calculated. Only the linear portion of each dissolution profile was considered for the intrinsic dissolution rate determination. The slope and the other statistical parameters of the curves were calculated by linear regression analysis.

### 3.10. Ultraviolet spectroscopy (UV-Vis)

UV-Vis spectra of VAL were registered on a HP UV/Vis, model 8453spectrophotometer (Hewlett Packard, Palo Alto, CA, USA), using 0.01 mol·L^-1^ NaOH as the solvent at a chosen wavelength of 225 nm. Quartz cuvettes with path length 1 cm were also used. The slope and the other statistical parameters of the calibration curves were calculated by linear regression analysis.

### 3.11. Anti-hypertensive evaluation

After evidence of VAL:β-CD inclusion formation, the antihypertensive efficacy *in vivo* was tested on VAL, VAL:β-CD complex prepared by the freeze-drying (FDY) and vehicle as control. The method used was telemetry, where male Wistar rats (14–16 weeks old) weighing 250–300 g were used. All rats were obtained from CEBIO (Centro de Bioterismo do Instituto de Ciências Biológicas, Universidade Federal de Minas Gerais). All rats in the study received subcutaneous infusion of angiotensin II (5 μg·h^-1^) throughout the experiment to induce hypertension. The rats were housed in separated cages, under controlled temperature conditions (25 °C) and 12/12 h light/dark cycle (light: 6:00 AM–6:00 PM), free access was allowed to standard diet (Nuvilav CR1 - Nuvital Nutrientes) and tap water was supplied *ad libitum*. Before the experiments, the animals underwent acclimatization for 7 days in an isolated telemetry room. The studies were performed in accordance with the guidelines for the human use of laboratory animals of our institution and approved by local authorities. A telemetry system (Data Sciences International, MN) was used for measuring mean arterial pressure (MAP). This monitoring system consists of a radio frequency transducer model TA11-PA C40, a receiver, a matrix, and an IBM-compatible personal computer with accompanying software (Dataquest A.R.T. Gold 2.0) to store and analyze the data. After insertion of an infusion pump containing the angiotensin II, the rats were housed in individual cages for three days until the telemetry tracing had indicated re-establishment of 24 h oscillations of blood pressure and heart rate. Data were sampled every 10 min for 72 h. After recovery, the rats were randomized in three experimental groups: vehicle (n = 4), FDY (n = 4) and VAL (n = 4). In order to characterize the cardiovascular effects of the inclusion compound in Wistar rats, we evaluated the variation in blood pressure after oral administration by gavage of the VAL (1.14 mg·kg^-1^ body weight) and the FDY inclusion complex in a dose of 4.22 mg·kg^-1^ body weight, equivalent at 1.14 mg of VAL. The period of observation was seven days after oral administration of control, VAL or FDY. Data were collected every 10 min during the entire experimental period. Time course: the variation in MAP was calculated each 10 min by the difference of the MAP value and an average of MAP values collected for 72 h before administration of the compounds. Data were analyzed by unpaired *t* test. All statistical analysis differences were considered significant at p < 0.05.

## 4. Conclusions

In our work different processes, namely FDY, KND and SDP, have been applied to prepare inclusion complex of VAL and β-CD. The phase solubility diagram was classified as A_L_-type indicating the formation of a 1:1 VAL:β-CD inclusion complex. The lower values of the stability constants (K_c_) suggest that the VAL:β-CD interaction is weak and also that the inclusion process is exothermic, defined as enthalpically driven and spontaneous.

The host:guest interaction was better characterized by the dipolar correlation in the 2D-ROESY contour map, where protons from the aromatic region of the drug present correlations with protons inside the β-CD cavity. Another advantage is the use of only water for the FDY preparation, allowing clean production of inclusion compounds between VAL and β-CD with elimination of organic solvents involved in the processes described in the literature.

The solubility characterization by intrinsic dissolution shows that the complexes studied have better rate solubility than free VAL, at least 16 times more for FDY and 2.4 times more for SDP. This solubility gain can improve the oral bioavailability of VAL. In studies with Wistar rats VAL administered alone shown significant reduction in arterial pressure for two days after oral administration. On the other hand, FDY produced a significant reduction in arterial pressure after forty minutes and was able to reduce arterial pressure for four days exceeding VAL.

## Figures and Tables

**Table 1:** Linear regression and Stability constant (K_c_) calculated from the system VAL:β-CD at absolute temperature of 298 K, 303 K and 310 K.

Temperature	Linear equation	Linearity (R)	Stability constant (K_c_) ± SD
298 K	y = 0.0685x + 0.0004	0.9960	165.4 M^-1^ ± 0.16
303 K	y = 0.0646x + 0.0005	0.9947	145.0 M^-1^ ± 0.08
310 K	y = 0.0636x + 0.0006	0.9960	111.3 M^-1^ ± 0.02

**Table 2:** Thermodynamic parameters for complexation of VAL:β-CD.

**∆*H* (kJ·mol^-1^)**	-24.90
**∆*S* (J·mol^-^1·K^-1^)**	-0.041
**∆*G* (kJ·mol^-1^)**	-12.45

**Table 3:** Linear regression, linearity and intrinsic dissolution for VAL and complexes prepared by solid dispersion (SDP) and freeze-drying (FDY).

	Linear equation	Linearity	Intrinsic dissolution (mg/min/cm^2^) (± RSD)
VAL	y = 0.2481x + 0.1183	0.9996	1.0 (± 1.2%)
SDP	y = 0.5940x - 1.1834	0.9985	2.4 (± 2.4%)
FDY	y = 4.1940x + 0.2000	0.9962	16.8 (± 3.1%)

RSD: Relative Standard Deviation.
